# High levels of viral repression, malnutrition and second-line ART use in adolescents living with HIV: a mixed methods study from Myanmar

**DOI:** 10.1186/s12879-020-04968-x

**Published:** 2020-03-20

**Authors:** Jillian Murray, Katherine Whitehouse, Janet Ousley, Elkin Bermudez, Theint Thida Soe, Adelene Hilbig, Kyi Pyar Soe, Phyu Ei Mon, Kyaw Tint Tun, Win Le Shwe Sin Ei, Joanne Cyr, Carole Deglise, Iza Ciglenecki

**Affiliations:** 1 Médecins Sans Frontières, Geneva, Switzerland; 2 Médecins Sans Frontières, Geneva, Myanmar; 3 Myanmar Ministry of Health, National AIDS Program, Naypyitaw, Myanmar

**Keywords:** ALHIV, Teen clubs, Adherence, Lipodystrophy, Qualitative, HIV/AIDS

## Abstract

**Background:**

Adolescents living with HIV/AIDS (ALHIV) are a particularly vulnerable but often overlooked group in the HIV response despite additional disease management challenges.

**Methods:**

All ALHIV (10–19 years), on ART for ≥6 months, presenting to care at a Médecins Sans Frontières (MSF) clinic in Myanmar from January–April 2016 were eligible for the quantitative study component (clinical history, medical examination, laboratory investigation). A subset of these respondents were invited to participate in qualitative interviews. Interviews and focus groups were also conducted with other key informants (care givers, clinicians).

**Results:**

Of 177 ALHIV, 56% (100) were aged 9–13 years and 77 (44%) were 14–19. 49% (86) had been orphaned by one parent, and 19% (33) by both. 59% (104) were severely underweight (BMI < 16). 47% presented with advanced HIV (WHO stage III/IV). 93% were virally supressed (< 250 copies/mL). 38 (21%) of ALHIV were on a second-line ART after first-line virological failure. Qualitative interviewing highlighted factors limiting adherence and the central role that HIV counsellors play for both ALHIV patients and caregivers.

**Conclusions:**

Our study shows good clinical, immunological, and virological outcomes for a cohort of Myanmar adolescents living with HIV, despite a majority being severely underweight, presenting with Stage III or IV illness, and the prevalence of comorbid infections (TB). Many treatment and adherence challenges were articulated in qualitative interviewing but emphasized the importance of actively engaging adolescents in their treatment. Comprehensive HIV care for this population must include routine viral load testing and social support programs.

## Background

The adolescent period (from 10 to 19 years) is associated with rapid physical and psychological development [1]. Adolescents living with HIV/AIDS (ALHIV) are a particularly vulnerable but often overlooked group in the HIV response, despite the fact that they have additional challenges managing their disease. They are sometimes born to HIV-positive parents and may have been orphaned, they do not always understand their disease status, and they must navigate a multitude of emotional, psychological, and physical changes [2]. Earlier anti-retroviral therapy (ART) initiation, good retention in care, and drug adherence are associated with higher CD4 counts, viral suppression, and lower mortality, yet evidence suggests that all of these are problematic in this age group [3–5].

Global projections anticipate the number of ALHIV will grow as perinatally infected children survive into adolescence and new horizontal infections start to occur [1]. As a result, it is increasingly important to understand the unique challenges ALHIV face in a variety of contexts and regions. We report the results of a mixed-methods study exploring the clinical characteristics, attitudes, and understanding of HIV in an ALHIV cohort in Southern Myanmar in order to inform clinical practice involving this age group. It is the first study to examine this patient population in the country that distinguishes young (10–13) from older (14–19) adolescents as unique subgroups.

## Methods

Since 2004, Médecins Sans Frontières (MSF) has been supporting free outpatient HIV testing and care in a rural community in south-eastern Myanmar. ALHIV receive specialized counselling and services to help them understand their diagnosis and treatment. Appointment scheduling occurs simultaneously with monthly teen groups that include sexual health education on HIV transmission and prevention. Annual adolescent mobilization days engage children and adolescents in fun, non-medical activities, facilitating friendships and establishment of a support network during their adolescent years.

Since 2014, MSF uses the World Health Organization (WHO) recommended approach to adolescent HIV management including regular viral load (VL) testing and enhanced adherence counselling (EAC) [6]. Paediatric formulations of abacavir (ABC), lamivudine (3TC), and efavirenz (EFV) are the preferred first-line treatment, with zidovudine (AZT), 3TC, and nevirapine (NVP) as the primary alternative choice. ART availability and protocols have changed over the years: stavudine (D4T)-based regimens were used until 2012, and Protease inhibitors (PI) have been available at MSF since 2007 but are reserved for second-line treatment. If a patient has consecutive detectable VL results following 3 months of EAC with documented good adherence, they are considered for second-line ART with presumed virological resistance. Genotyping is done for all patients with evidence of second-line failure.

This study had two components. All adolescent patients aged 10–19 years on ART for ≥6 months who presented during the study period (January–April 2016) were eligible for the quantitative component, including a clinical history, medical examination, and laboratory investigation conducted by a medical doctor and counsellor. A subset of those respondents were invited to participate in qualitative interviews with an HIV counsellor. These interviews were supplemented by additional interviewing and focus group discussions (FGD) with other key informants.

### Clinical history

A physical and neurological exam was conducted for each patient, and a laboratory investigation was conducted on whole blood samples. Study participants were asked about HIV transmission risk factors, treatment history, adherence, some sociodemographic characteristics, and their understanding of HIV and their status. A Patient Health Questionnaire A (PHQ-A), a modified for version of the PHQ-9, a standardized assessment tool, was used as a depression screening tool by a trained HIV counsellor.

### Medical and laboratory investigation

Lipodystrophy was based on a physician’s diagnosis of abnormal fat distribution (lipoatrophy or lipohypertrophy). Body mass index (BMI) scores were calculated as kg/cm^2^ and categorized using thresholds adjusted to WHO defined variations in Asian populations [7]. A 5-stage Tanner index assessed sexual maturity [8]. Laboratory investigation included a complete blood (CBC) and CD4 count, HIV VL, Hepatitis C virus (HCV) antibody and Hepatitis B surface antigen (HBsAg) testing, and measurement of cholesterol and triglyceride levels. An HIV VL of ≤250 copies/mL was considered undetectable. For patients with VL > 1000 copies/mL, HIV genotyping and resistance testing were completed at BiO Molecular Laboratory (Bangkok, Thailand). Hypercholesterolaemia was defined as cholesterol ≥240 mg/dL and hypertriglyceridemia as triglycerides ≥150 mg/dL*.*

### Qualitative study

In-depth interviews (IDIs) with 12 adolescents and 10 caregivers were conducted, and two focus group discussions (FGD) undertaken with MSF healthcare providers. Participants were purposively selected to achieve maximum sample heterogeneity including a variety of genders, geographic origins, and ages. IDI sample size was determined through an iterative process seeking a data “saturation point,” achieved when the study facilitators felt no original themes or new information were emerging from the interviews [9]. Interviews with all participants focused on adolescent treatment adherence and HIV status disclosure, with patients additionally asked about perceptions of quality of life, emotional state, and HIV knowledge. Interview questions about sexual practices differed between adolescents aged 10–13 and those 14–19 years, and were largely omitted for the younger group. IDIs with caregivers additionally enquired about patient support and family relationships, and healthcare provider FGDs were asking to consider emotional aspects of health. Data analysis was performed inductively using a Grounded Theory approach to develop a coding framework with the aid of NvivoPro 11.0 software [10].

### Statistical analysis

Retrospective cohort data was extracted from the MSF FUCHIA database (Follow-Up and Care for HIV/AIDS, v. 1.7 Epicentre, Paris, France) and analysed to determine characteristics of patients at baseline (treatment enrolment). Cross-sectional data were entered into Microsoft Excel. Baseline and descriptive data were calculated as proportions using Chi-squared or Fisher exact testing at a 5% significance level. Multiple logistic regression analysis investigated risk factors for lipodystrophy and hypertriglyceridemia. All statistical analyses were completed using Stata version 14.

Written and verbal informed consent was given by participants prior to enrolment, and guardians provided assent for adolescents < 18 years of age. The study proposal was approved by Ethics Review Committee at Department of Medical Research, Ministry of Health and Sports, Myanmar as well as the MSF Ethical Review Board. Approval was also obtained from the local medical authorities.

## Results

### Adolescent characteristics

A total of 177 adolescents were enrolled in the study, 54% (96) of whom were female (Table 1). Most adolescents reported perinatal HIV exposure as a risk factor for HIV transmission (115, 65%). (Table 1). Orphan status was common; with 86 (49%) orphaned by one parent, and 33 (19%) orphaned by both parents.

**Table 1:** Baseline demographic information adolescents aged 10–19 years old on ART and enrolled in the cross-sectional survey in Myanmar, by sex, 2016

Baseline Characteristics	Total (*n* = 177)	Female (*n* = 96)	Male (*n* = 81)	*P*-value
	# (%)	# (%)	# (%)	
Age (years)				0.058
9–13	100 (57)	48 (50)	52 (64)	
14–19	77 (43)	48 (50)	29 (36)	
Ethnicity				0.066
Bamar	156 (87)	85 (89)	69 (85)	
Karen	6 (3.4)	5 (5.2)	1 (1.2)	
Mon	13 (7.3)	6 (6.3)	7 (8.6)	
Other	4 (2.3)	0 (0.0)	4 (4.9)	
Presumed HIV exposure				0.043
Perinatal	115 (65)	60 (63)	55 (68)	
Blood transfusion	7 (4.0)	7 (7.3)	0 (0.0)	
Not identified	55 (31)	29 (30)	26 (32)	
Orphan				0.368
Not orphaned	58 (33)	34 (35)	24 (30)	
One parent	86 (49)	42 (44)	44 (54)	
Both parents	33 (19)	20 (21)	13 (16)	
Age of disclosure to adolescent				0.370
0–5	2 (1.1)	1 (1.0)	1 (1.2)	
6–9	47 (26)	25 (26)	22 (27)	
10–13	74 (41)	36 (37)	38 (47)	
14–19	14 (7.9)	7 (7.3)	7 (8.6)	
Missing	40 (22)	27 (28)	13 (16)	
Education level				0.522
Completed high school	10 (5.7)	6 (6.3)	4 (4.9)	
Attending school	117 (66)	60 (62)	57 (70)	
Not attending & not finished	50 (28)	30 (31)	20 (24)	
Occupation				0.000
Student	130 (73)	68 (71)	62 (77)	
Manual Worker	11 (6.2)	0 (0.0)	11 (14)	
Tailor	6 (3.4)	6 (6.3)	0 (0.0)	
Seller	5 (2.8)	5 (5.2)	0 (0.0)	
Unemployed	2 (1.1)	1 (1.0)	1 (1.2)	
Other	23 (13)	16 (17)	7 (8.6)	

Many (28%; 50) ALHIV were not attending or had not completed school, and many worked (12%, 22). Of those employed, 50% [11] of adolescent boys were manual labourers, and 50% [11] of adolescent girls were tailors or merchants. Almost no (< 1%) ALHIV displayed signs of depression using the adjusted PHQ-A methodology.

### HIV treatment characteristics

Half the ALHIV (92, 52%) first presented to the MSF clinic at ≤5 years old, with a mean time on any ART of 6.6 years (Table 2). Girls were significantly more likely than boys to present with a later HIV WHO Stage (*p* = 0.007). Overall, 47% (83) of adolescents presented to care with advanced HIV illness (defined as WHO stage III/IV illness), of which 20 had a CD4 < 200 copies/mL [11]. One-fifth (21%; 38) were on second-line ART after first-line virological failure. Eighty percent of adolescents aged 9–13 years and 77% of adolescents aged 14–19 years were on first-line treatment (Additional file 1). No patients were on a first-line PI-based regimen.

**Table 2:** HIV treatment data of adolescents aged 10–19 years old on ART and enrolled in the cross-sectional survey at Myittar Yeik Clinic, by sex, 2016

	Total	Female	Male	*P*-value
	# (%)	# (%)	# (%)	
Age (years) at ART start				0.513
<=5	92 (52)	45 (47)	47 (58)	
6–9	64 (36)	38 (40)	26 (32)	
10–13	14 (7.9)	9 (9.4)	5 (6.2)	
14–19	7 (4.0)	4 (4.2)	3 (3.7)	
WHO status at cohort entry				0.007
1	41 (23)	13 (14)	28 (35)	
2	52 (29)	32 (33)	20 (25)	
3	72 (41)	43 (45)	29 (36)	
4	11 (6.2)	8 (8.3)	3 (3.7)	
Missing	1 (0.6)	0 (0.0)	1 (1.2)	
CD4 count at cohort entry^†^				0.625
< 200	34 (19)	22 (23)	12 (15)	
200–350	14 (7.9)	6 (6.3)	8 (9.9)	
351–500	12 (6.8)	7 (7.3)	5 (6.2)	
> 500	65 (37)	33 (34)	32 (40)	
Missing	52 (29)	28 (29)	24 (30)	
Current ART regimen				0.090
First-line treatment	139 (79)	80 (83)	59 (73)	
Second-line treatment	38 (21)	16 (17)	22 (27)	
Mean time (years) on any ART regimen, SD	6.6 (2.7)	6.7 (2.7)	6.5 (2.4)	0.703
Mean time (years) on current ART regimen, SD	2.1 (1.0)	2.3 (1.0)	1.9 (0.8)	0.010

^†^Includes results up to 6 months after cohort entry

### Clinical characteristics

The majority (104, 59%) of adolescents were severely underweight (BMI < 16) (Table 3), though most had CD4 counts > 500 cells/μl (146, 82%) and were predominantly (165, 93%) virologically suppressed. Ninety-seven percent of adolescents aged 9–13 years were severely underweight compared to 61% of adolescents aged 14–19 years (*p* < 0.001) (Additional file 2). Adolescent girls were significantly more likely than boys to have a detectable VL (*p* = 0.05). Among the < 5% (*n* = 9) of adolescents with a VL > 1000 copies/mL, 71% (5/9) were on a second-line PI-based regimen following virological failure; 29% (4/9) remained on first-line ART despite evidence of inadequate virological suppression. 78% (7/9) demonstrated resistance mutations in their HIV genotypes (Table 4).

**Table 3:** Results of clinical and laboratory examinations, adolescents (10–19 years) on ART, by sex, Myanmar, 2016

	Total (*n* = 177)	Female (*n* = 96)	Male (*n* = 81)	*P*-value
	# (%)	# (%)	# (%)	
BMI				0.089
Severe underweight	104 (59)	49 (51)	55 (68)	
Moderate underweight	22 (12)	15 (16)	7 (8.6)	
Mild underweight	18 (10)	9 (9.4)	9 (11)	
Normal	32 (18)	22 (23)	10 (12)	
Normal (increased risk)	1 (0.6)	1 (1.0)	0 (0.0)	
CD4 count				0.827
< 200	1 (0.6)	1 (1.0)	0 (0.0)	
200–350	7 (4.0)	4 (4.2)	3 (3.7)	
351–500	23 (13)	14 (15)	9 (11)	
> = 500	146 (82)	77 (80)	69 (85)	
Viral load (copies/mL)				0.050
< 250	165 (93)	89 (93)	76 (94)	
250–1000	3 (1.7)	0 (0.0)	3 (3.7)	
> 1000	9 (5.1)	7 (7.3)	2 (2.5)	
Tanner Stage				0.007
1	63 (36)	23 (24)	40 (49)	
2	41 (23)	25 (26)	16 (20)	
3	32 (18)	19 (20)	13 (16)	
4	23 (13)	15 (16)	8 (10)	
5	18 (10)	14 (15)	4 (4.9)	
PHQ9 Score				1.000
Minimal depression	176 (99)	95 (99)	81 (100)	
Mild depression	1 (0.6)	1 (1.0)	0 (0.0)	
Moderate depression	0 (0.0)	0 (0.0)	0 (0.0)	
Co-morbidities and adverse effects				
Lipodystrophy	74 (42)	40 (42)	34 (42)	0.967
Neuropathy	7 (4.0)	3 (3.1)	4 (4.9)	0.704
Renal Insufficiency^a^	2 (1.1)	2 (2.1)	0 (0.0)	0.344
Hypercholesterolemia^b^	1 (0.6)	0 (0.0)	1 (1.2)	0.208
Hypertriglyceridemia^c^	32 (18)	17 (18)	15 (19)	0.761
HBsAG	3 (1.7)	2 (2.1)	1 (1.2)	1.000
Hepatitis C antibodies	1 (0.6)	1 (1.0)	0 (0.0)	1.000
History of opportunistic infections				
Pulmonary Tuberculosis	97 (55)	54 (56)	43 (53)	0.674
Extrapulmonary Tuberculosis	21 (12)	12 (13)	9 (11)	0.776
Oral esophageal candidiasis	6 (3.4)	5 (5.2)	1 (1.2)	0.221
Pneumocystis pneumonia	1 (0.6)	1 (1.04)	0 (0.0)	1.000
Disseminated non-TB mycobacteria	1 (0.6)	1 (1.04)	0 (0.0)	1.000

^*a*^*Renal insufficiency as creatinine clearance < 50 ml/min;*

^*b*^*Hypercholesterolemia as serum cholesterol ≥ 240 mg/dL;*

^*c*^*Hypertriglyceridemia as serum triglyceride level > 150 mg/dL;*

**Table 4:** Major and minor resistance mutations genotyped in seven adolescents with a VL > 1000 copies/mL, Myanmar, 2016

Adolescent	Current regimen	Type of mutation†		
		PI	NNRTI	NRTI
1	ABC.3TC.LPV/r	None	V179IT	None
2	TDF.3TC.LPV/r	L10IV	K101H, V179T, G190S	M41L, D67N, M184V, T215F
3	AZT.3TC.NVP	None	G190A	None
4	ABC.3TC.EFV	L10I	V106I, V179T, Y188L	None
5	ABC.3TC.LPV/r	None	G190A	M184V
6	ABC.3TC.LPV/r	None	Y181CY	None
7	TDF.3TC.LPV/r	None	K103, V108I, H221Y	M41L, D679, M184V, T215Y, K219R

†NNRTI=Non-nucleoside reverse transcriptase inhibitor; NRTI=Nucleoside reverse transcriptase inhibitor; PI=Protease inhibitor.

Nearly half (42%, 74) of adolescents had signs of lipodystrophy, which was associated with the use of a D4T-based regimen (Table 5) in regression analysis. Thirty-two (18%) adolescents had hypertriglyceridemia, which was associated with LPV and AZT use. Seven (4%) had neuropathy. Almost no adolescents had evidence of HBsAG (2%, 3) or HCV antibodies (< 1%, 1).

**Table 5:** Odds ratios for risk factors associated with lipodystrophy, Myanmar, 2016

	Lipodystrophy			Hypertriglyceridemia		
	OR (95% CI)	aOR (95% CI)	*p*-value	OR (95% CI)	aOR (95% CI)	*p*-value
Sex						
Female	ref	ref		ref	ref	
Male	1.0 (0.6–1.8)	0.80 (0.4–1.6)	0.589	1.1 (0.5–2.4)	0.9 (0.4–2.2)	0.768
Age (years) at ART start	0.8 (0.8–0.9)	0.9 (0.8–1.0)	0.059	1.0 (0.9–1.1)	1.1 (1.0–1.3)	0.067
WHO status at cohort entry						
1	ref	ref		ref	ref	
2	1.7 (0.7–4.0)	1.2 (0.4–3.1)	0.729	0.6 (0.2–1.8)	0.5 (0.2–1.5)	0.213
3	1.9 (0.9–4.3)	2.1 (0.8–5.4)	0.117	0.6 (0.2–1.5)	0.3 (0.1–1.0)	0.055
4	0.8 (0.2–3.6)	0.9 (0.2–5.0)	0.921	0.7 (0.1–3.7)	0.4 (0.1–2.8)	0.350
Exposure to D4T-based regimen^1^						
No	ref	ref		ref	ref	
Yes	19 (2.5–142)	11 (1.3–92)	0.028	5.5 (0.7–42)	8.1 (0.9–73)	0.061
Exposure to AZT-based regimen^1^						
No	ref	ref		ref	ref	
Yes	2.1 (1.1–4.2)	1.7 (0.6–4.3)	0.290	1.0 (0.4–2.4)	3.3 (1.0–10.3)	0.042
Exposure to PI-based (LPV) regimen^1^						
No	ref	ref		ref	ref	
Yes	2.0 (1.0–4.1)	1.3 (0.4–3.8)	0.645	3.7 (1.6–8.3)	7.7 (2.2–26.8)	0.001
Exposure to EFV-based regimen^1^						
No	ref	ref		ref	ref	
Yes	0.3 (0.2–0.7)	0.5 (0.2–1.3)	0.161	0.6 (0.3–1.4)	2.3 (0.7–7.4)	0.148

^1^*Exposure is considered at least six months of treatment with the drug*

Over half (55%; 97) of adolescents had a previous diagnosis of pulmonary tuberculosis, the most common opportunistic infection (OI) among this group, with an extrapulmonary TB diagnosis in a further 12% [12]. Other WHO Stage IV infections are detailed in Table 3.

### Differences between younger and older adolescents

One hundred ALHIV (57%) were aged 9–13 years and 77 (43%) were aged 14–19 years (Additional file 1). Among the older age group, 41 (53%) presented at WHO stage III or IV compared to 42 (42%) among the younger age group. Both age groups included 48 females, which corresponds to 48% of the younger age group and 62% of the older age group.

### Qualitative results

A total of 22 IDIs and 2 FGDs (with 6 to 8 participants per group), were conducted from January to April 2016. The results comprise multilevel thematic areas organized into four primary domains: the disclosure of HIV status to an adolescent, caregiver HIV knowledge and communication, adherence, and the social and emotional needs of adolescents.

### HIV status disclosure

ALHIV report becoming aware of their HIV status between 7 and 10 years of age, with varying degrees of understanding as they grow older. Clinic counsellors described a progressive disclosure process used by MSF to provide information according to young patients’ developmental levels. Some primary caregivers chose to delay disclosure, often relying on advice from clinic staff about when to do so. Furthermore, they preferred counsellors initiate the disclosure process due to their concerns about the ALHIV being too young to understand their disease and apprehension about their possible negative emotional response upon status disclosure.

ALHIV described networks of caregivers, with secondary or “back-up” caregivers in the absence of their primary caregiver or for specific functions. The caregiver role varied from supporting adolescents’ treatment adherence to attending clinic appointments, managing nutrition, and providing emotional and financial support. Around half of adolescents interviewed described widespread status disclosure (to other family members, neighbours or community members, school teachers, friends or peers and work colleagues) and others described selective disclosure to limited people. Caregivers maintained that social engagements which demanded taking medication in front of other people impeded treatment adherence.

### HIV knowledge and communication

Some primary caregivers experienced feelings of anger, guilt or responsibility. Clinic staff perceived these feelings as inhibiting good communication, health education, and positive relationships in the household, which they considered essential to adolescents’ continued engagement in HIV services. Yet very few adolescents reported ever having received health education from caregivers or family members, with the majority attributing health knowledge to clinic counselling staff.

*“Some parents … feel sadness when talking about their children. They assume the children have to take [medication] because of them. They don’t dare say something to child... But they need to explain to the child how to take the pills correctly. If not, there is a problem in taking [medication] if there is a misunderstanding between the parents and their children.”*

(Doctor, female, clinic staff, FGD 1)

Though some caregivers did display coherent knowledge of HIV management and treatment adherence, including the danger of missed or late dosing, most described insufficient health education or HIV literacy as a limitation to properly discussing HIV. Instead, they relied on the advice of clinical staff regarding the management of their adolescents. Counsellors were identified as playing a particularly central role to support caregiver communication both by clinic staff and caregivers. Clinic staff recommended that caregiver health education on HIV management be integrated into services.

### Drug adherence

Most participating adolescents were able to recall their type of medication and that it works by controlling rather than curing the virus. Some knew that medication should be taken daily, at specific times, for life. Many experienced the death of immediate or extended family members due to HIV which, when combined with their own HIV status, resulted in a strong awareness of the function of treatment in achieving good health, as well as of their own and others’ mortality.

“*… I explained to her that her mother died with this infection. I told her that she will live if she took the pills. Now she is surviving by taking them. She told me about it [ …*] *I replied to her, “You will live since you already have treatment for your diagnosis”. And she feels comfort after taking medication.”*

(Caregiver 3, grandfather of orphaned adolescent female living with HIV, in-depth interview)

Several challenges to managing HIV were cited by adolescents. Taking medication or attending clinic appointments were seen as interfering with education, work, social activities, or over-night trips. Caregivers had to emphasize the importance of treatment adherence over school or social commitments, and an interruption in a primary caregiver’s presence was identified as a critical moment when treatment adherence was more likely to lapse. Staff FGDs highlighted a common theme in adolescent populations: the emerging desire for privacy or freedom from familial networks. This was reported more in older adolescents, demonstrating their gradual transition from total dependence for HIV management towards greater autonomy and responsibility for self-care. Staff perceived that daily HIV treatment often seemed an irritating burden for adolescents and may lead to reluctance to adhere to treatment.

*“It is a duty for HIV patients because they are always taking pills … for life. It is annoying for them. And sometimes they don’t want to take the pills anymore.”*

(Nurse, female, clinic staff, FGD 1)

### Social and emotional needs of adolescents

The distinction of adolescent years from both childhood and adulthood with respect to emotional maturity and relationships was mentioned by many clinic staff and caregivers. These groups also understood the burden and responsibilities of life-long HIV treatment for young people. Some existing approaches to HIV management in adolescents, such as peer education and counselling sessions, were highly valued by adolescents interviewed, who maintained that meeting other ALHIV had positive impacts on their self-acceptance of and engagement with HIV services. FGDs revealed clinic staff’s strong support for adolescent adapted models of care. Counsellors central role was once again identified, engaging adolescents in services, providing them with health education, and supporting caregivers in the HIV status disclosure process.

*"Adolescent 8: Seeing peers who were [also] on treatment made me feel released that it was not only me, that others also have this disease.*

*Interviewer: Did you have this feeling for a long time?*

*Adolescent 8: After some time … seeing peers, I understood that it was not only me. I didn’t want to come to the clinic in the beginning. But now, I am ok and am not feeling uncomfortable anymore."*

(Adolescent number 8, female, in-depth interview)

## Discussion

Our study found that over 6 years of ART treatment, a cohort of adolescents in Myanmar maintained good clinical, immunological, and virological outcomes, despite a majority being severely underweight, half presenting late to care (with Stage III or IV illness), and in the face of the many treatment and adherence challenges articulated in qualitative interviewing with patients, caregivers, and clinicians. Notable differences occurred between genders, with adolescent girls more likely to present later to care and be viraemic, even after initiating ART.

Growth failure as a feature of HIV in children and adolescents is a concerning trend reported by other authors, though it is not often seen in paediatric and ALHIV populations in high-income countries [13]. The high proportion (59%) of severely underweight ALHIV in this cohort has implications for weight-based ART dosing in the Myanmar context, and highlights the importance of access to paediatric formulations. Of concern, a Ugandan study in children found malnutrition alters the bioavailability of EFV and LPV, and increases that of NVP, though bioavailability analysis was beyond the scope of this study [14].

The large majority (79%) of the Myanmar ALHIV cohort was on first-line treatment, slightly lower than other countries in the region (85%), but higher than a previous report of HIV treatment rates in adults and adolescents in Myanmar [15]. However one-fifth of the adolescents in our cohort had been switched to second-line ART (with presumed virological failure) [1]. This proportion is greater than were switched in prior ALHIV studies in other contexts and may partially be explained by the use of routine VL monitoring to establish failure, a feature often absent in other adolescent treatment programs that rely on WHO staging level or CD4 counts [16, 17].

Late presentation was a significant issue for these young people. Overall, 47% of the adolescents presented with advanced HIV and nearly half also presented with co-morbid infections (primarily TB). Parents and caregivers of adolescents in this cohort were waiting too long to access HIV care for their infected children, thus these children were often very sick on presentation to care. Late presentation is a global challenge for all age groups of people living with HIV and, as in Myanmar, is often due to expensive and scarce transport, missed work for caregivers, and HIV associated stigma [18, 19]. These Myanmar adolescents seem to do better over time than those in other regions, with much higher rates of viral suppression overall (93%) than adolescents in other studies, where high rates of virological failure among adolescents is common; in African contexts, adolescence has been established as a predictor of viraemia [4, 16]. Adolescents in Asia are generally more adherent to treatment than adolescents globally (83% vs 62%), and in qualitative interviews in our cohort, most adolescents expressed an understanding that missing doses of medication can lead to unsuccessful treatment [3, 20]. These encouraging rates of viral suppression are higher than even MSF’s own analysis from 2014 data, which found far more adolescents with detectable VL; 20% vs. the 7% identified here [21]. This previous analysis occurred before routine VL measurement and efforts to provide targeted support to adolescents began, and may underline the importance of these activities, though it is difficult to make direct comparisons.

Future research is warranted into the strong performance of Myanmar HIV adolescents on treatment, especially in other regions of the country and among those receiving care in facilities that do not offer the teen groups, VL monitoring, or counselling services that MSF provides. Qualitative analysis demonstrated that adolescents have positive attitudes towards the activities at the clinic and benefit from forming relationships with other ALHIV. The fact that only one adolescent reported recent symptoms of depression supports this. Peer education and social activities beginning from a young age may be protective factors for emotional health in adolescents, which could increase retention in care.

Far fewer of our participants (72%) were currently attending or had completed school than those in Thailand, Malaysia, Vietnam, Cambodia, where studies showed an average of 93% school attendance [3]. In interviews, Myanmar ALHIV who did attend school felt their education was impacted by the burden of HIV (taking treatment and attending the clinic) and caregivers emphasized that treatment adherence should be prioritized over school commitments. Additionally, though we found a higher rate of uneducated adolescents, most were also working, reflecting the high rates of child and adolescent labour in Myanmar generally [12]. Only 14% of our ALHIV cohort reported that they were not employed.

Finally, ART side effect profiles are well recognized. Abnormal physical changes, including lipodystrophy, have been linked to D4T, AZT, and to PIs (to a lesser extent), and can have an impact on body image in ALHIV and effects on treatment adherence [22]. Our findings are consistent with previous research demonstrating the established association between lipodystrophy and D4T, though our cohort was substantially more affected by this toxicity (42%) than ALHIV cohorts in other settings [23].

## Conclusion

Our study in a comprehensive HIV clinic in southern Myanmar found a large proportion of severely underweight adolescents and high levels of second-line ART use. Yet it also found a much lower proportion of virological failure when compared to other studies. This is likely a positive outcome of routine VL monitoring, following WHO treatment and ART switching guidelines, and ALHIV specific programs targeting adolescents’ social and mental health. A multidisciplinary approach that includes caregivers and adolescents as active agents in their own disease-management is desirable and feasible. Permitting families to speak openly in clinical, counselling, and group sessions will enhance communication skills and prepare adolescents to adopt responsibility for self-care. A broader sexual and reproductive health approach will facilitate adolescent growth, maturation, and will better prepare them for living with HIV as adults. Adolescent focused HIV programming can help young people adhere to their treatment and plan for their future as they bridge the gap between childhood and adulthood.

## Supplementary information

**Additional file 1.** Baseline characteristics of adolescents aged 10–19 years old on ART and enrolled in the cross-sectional survey at an MSF clinic in Myanmar, by age group.

**Additional file 2.** Results of the clinical examination of adolescents aged 10–19 years old on ART and enrolled in the cross-sectional survey at an MSF clinic in Myanmar, by age group.

## Data Availability

The datasets generated by this study are not publicly available because of the sensitive and confidential nature of data regarding HIV-positive adolescents.

## Abbreviations

ABC: Abacavir
ALHIV: Adolescents living with HIV/AIDS
ART: Anti-retroviral therapy
BMI: Body mass index
CBC: Complete blood count
EFV: Efavirenz
FGD: Focus group discussions
HBsAg: Hepatitis B surface antigen
HCV: Hepatitis C virus
HIV: Human immunodeficiency virus
IDIs: In-depth interviews
3TC: Lamivudine
MSF: Médecins sans frontières
NVP: Nevirapine
OI: Opportunistic infection
PHQ-A: Patient health questionnaire A
PI: Protease inhibitors
D4T: Stavudine
VL: Viral load
WHO: World health organization
AZT: Zidovudine
