# Supplementary material for: High levels of viral repression, malnutrition and second-line ART use in adolescents living with HIV: a mixed methods study from Myanmar
Source: BMC Infect Dis. 2020 Mar 20;20:241. doi: 10.1186/s12879-020-04968-x (PMC7085147; doi:10.1186/s12879-020-04968-x)
Supplement: Supplementary file 1 — Additional file 1. Baseline characteristics of adolescents aged 10–19 years old on ART and enrolled in the cross-sectional survey at an MSF clinic in Myanmar, by age group. [file 12879_2020_4968_MOESM1_ESM.docx]

**Additional File 1**

Additional file 1. Baseline characteristics of adolescents aged 10-19 years old on ART and enrolled in the cross-sectional survey at an MSF clinic in Myanmar, by age group

|  | Total  (n=177) | | 9-13 years  (n=100) | | 14-19 years  (n=77) | | P-value |
| --- | --- | --- | --- | --- | --- | --- | --- |
|  | # | % | # | % | # | % |  |
| Sex |  |  |  |  |  |  | 0.058 |
| Female | 96 | 54 | 48 | 48 | 48 | 62 |  |
| Male | 81 | 45 | 52 | 52 | 29 | 38 |  |
| WHO status at cohort entry |  |  |  |  |  |  | 0.307 |
| 1 | 41 | 23 | 25 | 25 | 16 | 21 |  |
| 2 | 52 | 29 | 33 | 33 | 19 | 25 |  |
| 3 | 72 | 40 | 35 | 35 | 37 | 48 |  |
| 4 | 11 | 6.2 | 7 | 7.0 | 4 | 5.2 |  |
| Missing | 1 | 0.6 | 0 | 0.0 | 1 | 1.3 |  |
| Age (years) at cohort entry |  |  |  |  |  |  | <0.001 |
| <=5 | 92 | 51 | 69 | 69 | 23 | 29 |  |
| 6-9 | 64 | 36 | 28 | 28 | 36 | 46 |  |
| 10-13 | 14 | 7.9 | 3 | 3.0 | 11 | 14 |  |
| 14-19 | 7 | 3.9 | 0 | 0.0 | 7 | 9.1 |  |
| CD4 count at cohort entry |  |  |  |  |  |  | 0.004 |
| <200 | 34 | 19 | 14 | 14 | 20 | 25 |  |
| 200-350 | 14 | 7.9 | 7 | 7.0 | 7 | 9.1 |  |
| 351-500 | 12 | 6.8 | 5 | 5.0 | 7 | 9.1 |  |
| >500 | 65 | 36 | 49 | 49 | 16 | 20 |  |
| Missing | 52 | 29 | 25 | 25 | 27 | 35 |  |
| Time (years) on ART |  |  |  |  |  |  | 0.002 |
| 0-4 | 51 | 29 | 37 | 37 | 14 | 18 |  |
| 5-8 | 68 | 38 | 40 | 40 | 28 | 36 |  |
| >8 | 58 | 33 | 23 | 23 | 35 | 45 |  |
| Current ART regimen |  |  |  |  |  |  | 0.588 |
| First-line treatment | 139 | 79 | 80 | 80 | 59 | 77 |  |
| Second-line treatment | 38 | 21 | 20 | 20 | 18 | 23 |  |
