# Supplementary material for: High levels of viral repression, malnutrition and second-line ART use in adolescents living with HIV: a mixed methods study from Myanmar
Source: BMC Infect Dis. 2020 Mar 20;20:241. doi: 10.1186/s12879-020-04968-x (PMC7085147; doi:10.1186/s12879-020-04968-x)
Supplement: Supplementary file 2 — Additional file 2. Results of the clinical examination of adolescents aged 10–19 years old on ART and enrolled in the cross-sectional survey at an MSF clinic in Myanmar, by age group. [file 12879_2020_4968_MOESM2_ESM.docx]

**Additional file 2**

Additional file 2. Results of the clinical examination of adolescents aged 10-19 years old on ART and enrolled in the cross-sectional survey at an MSF clinic in Myanmar, by age group

|  | Total  (n=177) | | 9-13 years  (n=100) | | 14-19 years  (n=77) | | P-value |
| --- | --- | --- | --- | --- | --- | --- | --- |
|  | # | % | # | % | # | % |  |
| BMI |  |  |  |  |  |  | <0.001 |
| <18.5 | 144 | 81 | 97 | 97 | 47 | 61 |  |
| 18.5-25 | 33 | 189 | 3 | 3.0 | 30 | 39 |  |
| CD4 count |  |  |  |  |  |  | 0.001 |
| <200 | 1 | 0.6 | 1 | 1.0 | 0 | 0.0 |  |
| 200-350 | 7 | 4.0 | 1 | 1.0 | 6 | 7.8 |  |
| 351-500 | 23 | 13 | 7 | 7.0 | 16 | 21 |  |
| >=500 | 146 | 82 | 91 | 91 | 55 | 71 |  |
| Viral load (copies/mL) |  |  |  |  |  |  | 0.172 |
| <250 | 165 | 93 | 90 | 90 | 75 | 97 |  |
| 250-1000 | 3 | 1.7 | 3 | 3.0 | 0 | 0.0 |  |
| >1000 | 9 | 5.1 | 7 | 7.0 | 2 | 2.6 |  |
| Tanner Stage |  |  |  |  |  |  | <0.001 |
| 1 | 63 | 36 | 59 | 59 | 4 | 5.2 |  |
| 2 | 41 | 23 | 26 | 26 | 15 | 19 |  |
| 3 | 32 | 18 | 12 | 12 | 20 | 25 |  |
| 4 | 23 | 13 | 3 | 3.0 | 20 | 26 |  |
| 5 | 18 | 10 | 0 | 0.0 | 18 | 23 |  |
| PHQ9 Score |  |  |  |  |  |  | 0.565 |
| Minimal depression | 176 | 99 | 99 | 99 | 77 | 100 |  |
| Mild depression | 1 | 0.6 | 1 | 1.0 | 0 | 0.0 |  |
| Moderate depression | 0 | 0.0 | 0 | 0.0 | 0 | 0.0 |  |
| Clinical manifestations |  |  |  |  |  |  |  |
| Lipodystrophy | 74 | 41 | 41 | 41 | 33 | 43 | 0.804 |
| Neuropathy | 7 | 4.0 | 3 | 3.0 | 4 | 5.2 | 0.470 |
| Renal Insufficiency^1^ | 2 | 1.1 | 2 | 2.0 | 0 | 0.0 | 0.328 |
| Hypercholesterolemia^2^ | 1 | 0.6 | 1 | 1.0 | 0 | 0.0 | 0.682 |
| Hypertriglyceridemia^3^ | 32 | 18 | 19 | 19.0 | 13 | 17 | 0.615 |
| History of opportunistic infections |  |  |  |  |  |  |  |
| Pulmonary Tuberculosis | 97 | 55 | 55 | 55 | 42 | 55 | 0.952 |
| Extrapulmonary Tuberculosis | 21 | 12 | 12 | 12 | 9 | 12 | 0.949 |
| Oral esophageal candidiasis | 6 | 3.4 | 2 | 2.0 | 4 | 5.2 | 0.406 |
| Pneumocystis pneumonia | 1 | 0.6 | 0 | 0.0 | 1 | 1.3 | 0.435 |
| Disseminated non-TB mycobacteria | 1 | 0.6 | 1 | 1.0 | 0 | 0.0 | 1.000 |

1. Renal insufficiency as creatinine clearance < 50 ml/min;
2. Hypercholesterolemia as serum cholesterol ≥240 mg/dL;
3. Hypertriglyceridemia as serum triglyceride level >150 mg/dL
